# Separation of spermatozoa from erythrocytes using their tumbling mechanism in a pinch flow fractionation device

**DOI:** 10.1038/s41378-019-0068-z

**Published:** 2019-05-20

**Authors:** Johanna T. W. Berendsen, Jan C. T. Eijkel, Alex M. Wetzels, Loes I. Segerink

**Affiliations:** 1 BIOS Lab on a Chip Group, MESA+Institute for Nanotechnology, Technical Medical Centre, University of Twente, Enschede, The Netherlands; 2 Department of Obstetrics and Gynaecology, Radboud University Nijmegen Medical Centre, Nijmegen, The Netherlands

**Keywords:** Microfluidics, Nanobiotechnology

## Abstract

Men suffering from azoospermia can father a child, by extracting spermatozoa from a testicular biopsy sample. The main complication in this procedure is the presence of an abundance of erythrocytes. Currently, the isolation of the few spermatozoa from the sample is manually performed due to ineffectiveness of filtering methods, making it time consuming and labor intensive. The spermatozoa are smaller in both width and height than any other cell type found in the sample, with a very small difference compared with the erythrocyte for the smallest, making this not the feature to base the extraction on. However, the length of the spermatozoon is 5× larger than the diameter of an erythrocyte and can be utilized. Here we propose a microfluidic chip, in which the tumbling behavior of spermatozoa in pinched flow fractionation is utilized to separate them from the erythrocytes. We show that we can extract 95% of the spermatozoa from a sample containing 2.5% spermatozoa, while removing around 90% of the erythrocytes. By adjusting the flow rates, we are able to increase the collection efficiency while slightly sacrificing the purity, tuning the solution for the available sample in the clinic.

## Assisted Reproductive Technology: Separating sperm cells from red blood cells

To assist men lacking spermatozoa (sperm cells) in the ejaculate (a condition called azoospermia) in fathering a child, a method has been developed to separate spermatozoa from erythrocytes (red blood cells). With azoospermia, a biopsy is performed to extract spermatozoa, which are used for intracytoplasmic sperm injection (a sperm is directly injected into the egg). The main complication with this procedure has been the abundance of erythrocytes, which have to be manually separated owing to the ineffectiveness of filtering methods. A team headed by Loes I. Segerink at the University of Twente, the Netherlands devised a technique for separating the two cell types using the tumbling behavior of spermatozoa, which is based on their length being about five times greater than the diameter of erythrocytes. The authors were able to extract 95% of spermatozoa while removing around 90% of erythrocytes.

## Introduction

Assistive Reproductive Technology is used all over the world to help couples with fertility problems conceive. It is estimated that 1 in 20 males has a low sperm count^1^, which causes fertility problems and often necessitates outside help in the form of a spermatozoa selection and subsequent intrauterine insemination or in vitro fertilization. If the sperm count is very low, intracytoplasmic sperm injection (ICSI) might be necessary. In more rare cases (~1 in 100 males), there are no spermatozoa in the ejaculate (azoospermia), making it necessary to perform a biopsy for obtaining spermatozoa^2^. This method is called testicular sperm extraction (TESE), after which the spermatozoa are extracted from the biopsy sample and used in ICSI^2^. Azoospermia can be split into two classes: obstructive and non-obstructive. In the first class, the cause for the lack of spermatozoa in the ejaculate is due to an obstruction in the reproductive tubes that prevents the spermatozoa from ending up in the ejaculate. In the second class, there is no obstruction, but there is a problem with the production of spermatozoa. This means that even in a biopsy sample, the sperm count is very low.

During a TESE, samples that are obtained from a patient with non-obstructive azoospermia contain only a very small number of spermatozoa, submerged in a mixture full of other tissue cells, other compounds, and a large amount of blood cells that are introduced due to blood vessel damage. The largest tissue cells and leukocytes are currently filtered out, e.g., by density gradient centrifugation, after which the remaining sample, containing mainly erythrocytes and spermatozoa, is imaged under a microscope. The sample, typically containing very few spermatozoa (<1%), is then searched for spermatozoa, which is a time-consuming manual process and can take many hours depending on the available numbers of suitable spermatozoa and oocytes^2^. Moreover, the selected spermatozoa can differ in quality, thereby reducing fertilization and pregnancy rates. Some techniques have been introduced to better select spermatozoa for ICSI, such as the hyaluronic acid-binding assay^3^ and intracytoplasmic morphologically selected sperm injection^4^. Both techniques are not fully validated and therefore their value for improving the outcome of ICSI is doubtful. The development of a technique that reduces TESE sperm processing time in combination with sperm quality selection is our ultimate goal, which will lead to a next step in more efficient and effective fertility care.

Until now, our focus was on individual trapping of sperm for analysis using chip technology^5,6,7^ for a better quality selection in single sperm for ICSI. Although this research is still running, we started the second part: more efficiency in TESE work-up. In first instance, we are focusing on the separation of spermatozoa from erythrocytes. Finally, we will bring these two parts (the faster TESE work-up and the single sperm selection) together in developing a microfluidic chip that automatically selects the highest quality spermatozoa for ICSI.

The large amount of cells in the sample generated by the TESE procedure requires a microfluidic separation method that can tolerate a large sample throughput (billions of cells of very different sizes from ~2 to ~40 μm) without clogging. The standard filter types either show a lot of clogging (dead end filters)^8^ or result in the loss of a significant percentage of the initial sample or low purity (cross flow filters)^9^. As the spermatozoa in a testicular biopsy might be not developed enough to showcase swimming, we cannot rely on this phenomenon for separation, requiring an approach that uses an imposed flow. Son et al.^10^ reported being able to separate spermatozoa from erythrocytes in their work, where they used a spiral channel to separate spermatozoa. In their reported results, one can see that the spermatozoa are broadly distributed in the channel. Their method could not get high purity of the spermatozoa due to this effect without using multiple steps and sacrificing retention. Liu et al.^11^ used a pinch flow device to separate epithelial cells from spermatozoa for forensic purposes. They noticed poor focusing of the spermatozoa in the channel, but due to the large difference in size between epithelial cells (50 µm diameter) and spermatozoa (50 µm in length, but <6 µm in width), the results were good enough for this application. Their sample contained 30% spermatozoa and their final purity was 94.0 ± 4.7%, with a retention rate of 41.1 ± 2.9%. The poor focusing of spermatozoa in the pinch flow device is a problem for the separation of particles that are slightly bigger than spermatozoa, but we think this phenomenon can be used for the separation spermatozoa from of smaller particles. Therefore, we propose to use pinch flow fractionation (PFF) for the separation of spermatozoa from erythrocytes.

The dimension of erythrocytes are typically 7.5–8.7 μm and 1.7–2.2 μm in diameter and height, respectively^12^. In this work, boar spermatozoa are used as a model for human spermatozoa and these are 45 μm long, 4 μm wide at the head, and have a height of 1 μm^13^. In comparison, human spermatozoa are 55 μm long, 3 μm wide at the head, and have a height of 1 μm^14^. This means that the cell sizes in a testicular biopsy, especially that of erythrocytes, are comparable with that of a spermatozoon and therefore the long shape of the spermatozoon needs to be utilized. It is mentioned by Samuel et al.^15^, in their review on the possible usage of microfluidics for TESEs, that PFF is not ideal for the separation of particles of multiple sizes when compared with other techniques, although the non-focusing of the spermatozoa, caused by their shape, can offer us an advantage when using PFF. In our application, a PFF device allows for the retention of most particles, while preventing clogging, as it uses hydrodynamic methods to sort the particles instead of steric hindrance.

In this study we show that PFF can used to retrieve the few present spermatozoa from a testicular sample, by making use of the typical shape of spermatozoa.

## Results and discussion

### Particle distribution in the separation channel

**Fig. 1:**
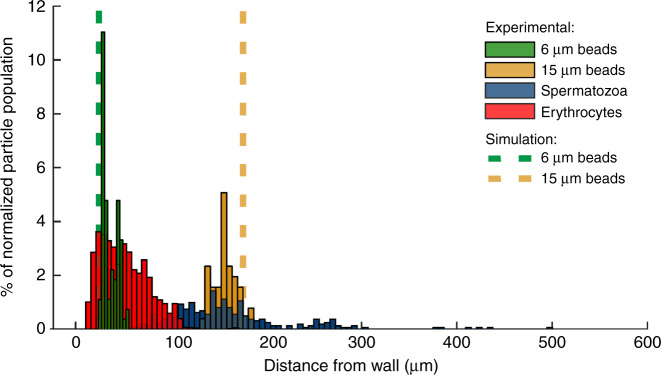
Separation in a PFF chip for different particle types. Example of a separation in a single chip with positions in the channel for different particle types: theoretical values for 6 and 15 µm beads have been obtained via Comsol simulations. Experimental values for 6 and 15 µm beads, as well as spermatozoa and erythrocytes, are included. Distance from the top wall (the sample side of the device) as measured at the center of the particle (head for spermatozoa)

The observed behavior of the beads in the microfluidic chip showed good agreement with our model in Comsol of beads traveling through an identical geometry. The observed behavior of spermatozoa however deviates from that of spherical beads in three noteworthy ways. First, the spermatozoa appeared in a broadened section with, on average, a larger distance from the wall than expected from their width (1–3 µm). Second, the distribution of the spermatozoa in the broadened section is also considerably wider than that of round particles (Fig. 1). Finally, spermatozoa that are parallel to the flow lines seem to end up closer to the wall than spermatozoa that are oriented perpendicular (Supplementary Fig. S1).

### Tumbling behavior of spermatozoa in PFF

**Fig. 2:**
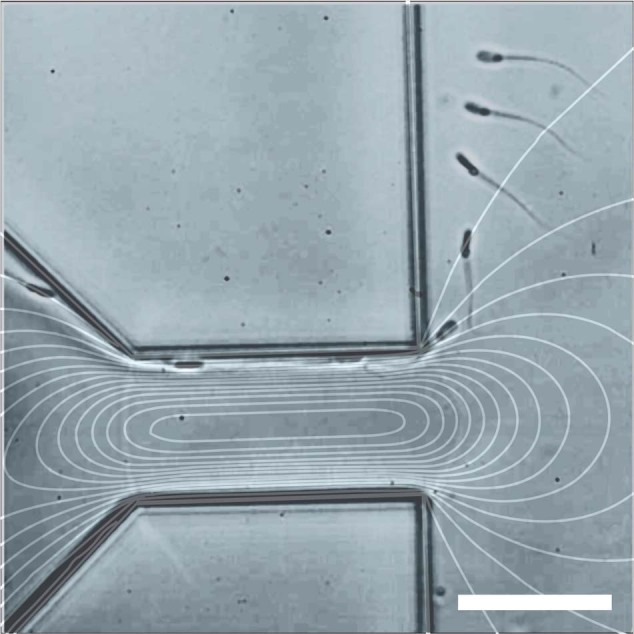
Tumbling behaviour of spermatozoa in PFF. Equivelocity lines and a spermatozoon passing through the pinched section. The tail occupies a part of the fluid that has a higher velocity than the part in which the head resides. The tail then gets pushed faster around the corner, causing a rotation in the *x*–*y* plane. Scale bar is 50 µm

The reason for the broad distribution of spermatozoa is the tumbling behavior that spermatozoa exhibit. Tumbling has previously been observed for many non-spherical particles^16^, including *Escherichia coli*^17^ and erythrocytes^18^. The rotation of non-spherical particles has also been used to aid in the hydrodynamic filtration of yeast cells^19^. Erythrocytes are also mentioned as not only exhibiting this type of motion but also a multitude of other motions due to their flexibility^18^. Spermatozoa are not axisymmetric (width vs. length and head vs. tail) and, therefore, also showcase tumbling behavior in a shear flow. Due to the large aspect ratio, the spermatozoa will mostly be oriented in their equilibrium orientation, which is aligned with the flow, either backwards or forwards depending on the vertical position in the channel^20,21^. However, in the PFF device, they are forced out of their equilibrium orientation due to the compressing and widening of the streamlines, inducing a tumble in the transition from the pinched section to the broadened section (Fig. 2).

Two forms of tumbling have been seen in the PFF device. The first form occurs in the *x*–*z* plane on the device, when the spermatozoa are forced to the wall of the pinched section (as they are “pinched” in the *y* direction). The high shear then causes them to tumble^20^. The second form of tumbling is mostly in the *x*–*y* plane of the device when the streamlines broaden after the pinch. The latter has an influence on the final separation process in the chip. This rotation of the spermatozoa happens at a critical time point in the device, when the streamlines are diverging and carrying particles of different sizes along their respective streamlines. PFF fractionation works with the principle that particles are forced into a specific streamline at the pinched section based on their size and will follow this line when the streamlines diverge, separating particles of different sizes. However, as the tail of a spermatozoon is deformable, but not so flexible that it can completely follow the streamline the head is occupying, the effective diameter of the spermatozoa will be larger. For the spermatozoon visible in Fig. 2, we see that the rotation causes the tail to be in a faster streamline (the lines in the figure give points of equal velocity, with the lines closer to the wall being slower than the lines in the center of the channel), which enhances the rotation even further. In the broadened section, the shear is much reduced due to the suddenly large width compared with the height of the channel. This slows down the tumbling and causes the spermatozoa to end up at a semi-random angle to the flow lines. The spermatozoa still feel some shear and will continue to rotate slowly as the spermatozoon is carried along the different streamlines until it aligns itself with the flow. This effect takes place on a much longer time scale than the separation that takes place due to the position of the particles in a certain streamline. The effect on the separation is negligible, as the flow lines in the broadened channel are parallel, and not diverging anymore, leading to no further change in the position relative to the channel wall. The apparent hydrodynamic radius of a spermatozoon can therefore lie anywhere between 1 and 50 µm (a video of several spermatozoa rotating can be found in the Supplementary Information). In our experiments, we observed that these extreme values do not appear, as the tail is not infinitely flexible, and the spermatozoon rotates in the transition of pinched to broadened section, and therefore does not appear already perpendicular to the flow rate when the flow lines start to diverge.

This larger effective diameter due to rotation makes the separation of the spermatozoa from larger cells more difficult. However, it is an advantage for the separation from cells that have equal smallest dimensions, but do not have such a large aspect ratio. Normally, a filter cannot be used for these separations, as a filter is based on the smallest dimension of a particle. Although erythrocytes also show tumbling behavior, the dispersion of these cells is less, as the larger axis of an erythrocyte is approximately one-fifth of that of a spermatozoon and therefore they will appear closer to the wall of the device. This tumbling phenomenon in the PFF device can therefore be used to separate spermatozoa from the abundant erythrocytes that reside in a typical biopsy sample.

### Separation of spermatozoa from erythrocytes

We have shown that due to the tumbling behavior of spermatozoa, these cells end up at a different position in the broadened channel than erythrocytes. The next step is to show that we are also able to isolate the spermatozoa from the sample.

**Fig. 3:**
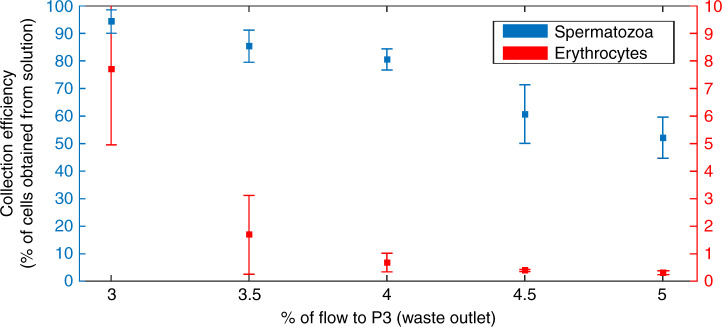
Collection efficienty of the PFF device. Collection efficiency of spermatozoa (blue) and erythrocytes (red) for different fluid removal ratios. With higher percentage of flow to outlet 3, fewer of both cell types are collected in outlet 4. However, by increasing the flow to outlet 3 with respect to outlet 4, the erythrocytes are more strongly excluded than spermatozoa. Error bars = 1 SD, *N* = 3

**Fig. 4:**
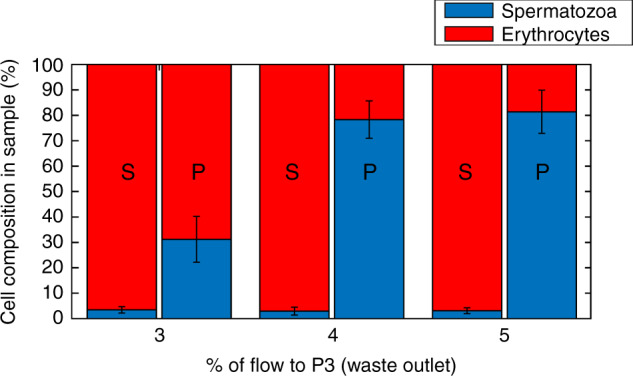
Extraction purity of the samples. Extraction purity of the samples. Sample compositions before and after separation for different fluid removal ratios (S = sample, P = product after separation). Error bars = 1 SD, *N* = 3

A whole blood sample, spiked with 2.5% spermatozoa, is made to mimic the composition of a testicular biopsy after gradient filtration. The 2.5% in the sample has been chosen to have enough spermatozoa to be able to do statistics on the results. This sample is running through the system, while the percentage of flow to the waste outlet (P3) is varied between 3% and 5%. If 3% of the total liquid is removed from the sample after the expansion, most spermatozoa are retained, but also a considerable percentage of the erythrocytes is kept in the collected sample. When larger percentages are removed, the amount of erythrocytes that are not separated out goes down dramatically. However, this also causes a larger percentage of spermatozoa to be removed from the sample (Fig. 3). The reason for this can also be observed in the position plot (Fig. 1), where the bands of the spermatozoa and erythrocytes overlap slightly. Figure 4 shows the sample composition for three different experiments. On the left, we see the composition for the initial sample; on the right, the composition of the obtained sample from the device is visible. It can be seen that even though at 3% fluid withdrawal, where the largest amount of spermatozoa are kept, the amount of erythrocytes in the obtained sample still is much larger than the amount of spermatozoa. The enrichment ratio (ER) of this experiment is ~10, yet the fraction of erythrocytes is still ~70%. In the experiments with 4% and 5% fluid withdrawal, the amount of erythrocytes in the final sample is much reduced and the ER is ~26. Due to the overlapping bands however, 100% extraction purity (EP) cannot be combined with 100% collection efficiency (CE), making it necessary to decide on the relative importance of the two parameters. Eighty-eight percent (±6%, *n* = 3) of the spermatozoa are viable after our separation and we can improve this by shorting the tubing, as this causes most of the damage to the cells. In our experiments, we have also discounted the presence of leukocytes. These make up a small percentage of the sample (<0.2%) and might have been in our product outlet, which would reduce the purity ratio. For simplicity in our experiments, we have not classed these as a separate species from erythrocytes. For comparison with the results from Liu et al.^11^ who has a CE of 41.1 ± 2.9% and an EP of 97.0 ± 2.3% for separation of spermatozoa from epithelial cells of 50 µm, we have obtained higher CEs, but lower EPs, with a CE higher than 52 ± 0.3% for an EP of 81 ± 8%, and up to over 94 ± 8% for an EP of 31 ± 9%. This is due to the different application, as we are not looking for identification of a subject but for the harvesting of spermatozoa for fertilization. Next to this, Liu et al.^11^ separated spermatozoa from epithelial cells that were 50 µm in diameter, whereas we separate spermatozoa from erythrocytes, which have a very similar smallest dimension.

Different sample types require different output specifications. Samples with a larger amount of spermatozoa can be set to remove more of the erythrocytes, in the process losing some of the spermatozoa as well. For samples with a lower sperm count, one can settle for a lower removal rate, but to keep a larger fraction of the spermatozoa. For the final application, a careful consideration is needed to find a balance between the EP and the ER. The system can be tuned to obtain a sample with more spermatozoa (but less pure) or less spermatozoa in a purer sample. This is dependent on the wishes of the clinician, who decides on an estimate of the amount of spermatozoa that are available in the total sample. This estimate is made during diagnosis of the severity of the condition and is known before the biopsy sample is processed. With this estimate, it is possible to choose the percentage of liquid to be directed toward the waste outlet (adjusting the pressure setting of the pump according to our script), with more liquid to be removed if one wants a purer sample and less removal of liquid if one wishes for a large amount of the spermatozoa to be retained.

## Conclusion

Due to the tumbling behavior of spermatozoa in a pinched flow, we are able to retrieve the spermatozoa from the erythrocytes in a mock sample of a testicular biopsy. By adjusting the flow rates, a high CE can be achieved for samples with very few spermatozoa (<20). We are able to obtain 95% of the spermatozoa, while removing ~90% of the erythrocytes. In samples with more spermatozoa, a high CE can also be sacrificed for a higher purity. They can choose to collect 85% of the spermatozoa, while removing 99% of the red blood cells to make the final selection of spermatozoa easier for the final application.

## Materials and methods

### Chip design

**Fig. 5:**
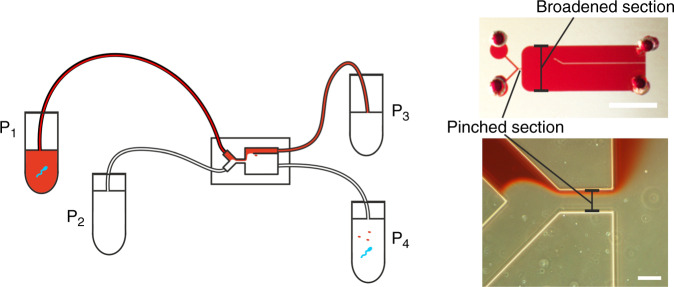
Set-up with microfluidic chip. Left: outline of the setup, features are not true to size. P1 is the sample pressure, P2 is the buffer pressure, P3 is the waste outlet pressure, and P4 is the product outlet pressure. The chip contains a pinched section and a broadened section. The cells get pushed toward the wall in the pinched section and appear at a distance from the top wall in the broadened section according to their apparent hydrodynamic radius. This distance determines the outlet that the cells will go through. Right, top: PFF chip. Scale bar is 2.5 mm. Bottom: visualization of the flow in the chip using red dye. Scale bar is 50 µm

The microfluidic chip consists of a PFF design, containing two inlets (width: 50 µm): one for the sample and one for the buffer joining into a single channel (the pinched section) that is 50 µm wide and 125 µm long (see Fig. 5). The channel then broadens abruptly to 2500 µm and splits into two outlets. The chip has a channel depth of 50 µm and was designed using CleWin software (version 5.0.12.0). Master molds for polydimethylsiloxane (PDMS) fabrication were produced by standard photolithography. Chips were fabricated using PDMS (Sylgard 184, Dow Corning, Midland, MI, USA) in a 1:10 v/v ratio of base vs. curing agent. PDMS and glass are used, as they are nontoxic to the spermatozoa. The PDMS was poured onto a silicon wafer, degassed, and cured at 60 °C overnight. After curing, microfluidic inlets and outlets were punched using Harris UniCore punchers (tip ID 1.0 mm, Ted Pella, Inc., Redding, CA, USA). The chips were bonded to glass microscope slides after activation by oxygen plasma using a plasma cleaner (model CUTE, Femto Science, Hwaseong-Si, South Korea).

### Sample preparation

To mimic a pre-filtered biopsy sample, as typically found in the clinic, we use a sample that consists of whole blood, spiked with boar spermatozoa, which is diluted five times. Fresh boar semen was obtained from a local artificial insemination center (“KI Twenthe”, Fleringen, The Netherlands) at a concentration of 20 × 10^6^ cells ml^−1^. The whole blood sample was obtained with informed consent from the donors from the Experimental Center for Technical Medicine (TechMed Centre, University of Twente, Enschede, The Netherlands). The erythrocytes were counted in the blood samples (~5 × 10^9^ cells ml^−1^) and the sample was mixed with spermatozoa containing solution to end up with 2.5% of spermatozoa in the sample. This concentration is slightly higher than in clinical samples, to obtain a better fidelity for the analysis. The sample was then diluted five times for handling with Beltsville Thawing Solution (BTS, Solusem, Aim Worldwide, Vught, The Netherlands).

### Experimental setup

Two sets of experiments are carried out. The first set is to characterize the behavior of spermatozoa and erythrocytes after PFF. The second set is to show the efficiency of spermatozoa isolation from a sample containing erythrocytes. For all these experiments, the same chip geometry is used, while changing the outflow ratios. The chip was connected to a Fluigent pump (Fluigent, Paris, France) using four channels and four equal length in- and outlet tubing. The fluidic resistances of the channels in the chip were calculated and measured with stagnation pressures. The in- and outlet pressures (P1–4) were then adjusted for a flow rate ratio of 1:20 (sample flow/total flow) for the inlets and 3/3.5/4/4.5/5:100 (waste/total) for the outlets. For this we used a Matlab script (Matlab 2015b, Mathworks, Natick, MA), which translated the wanted flow percentages to input pressures for the pressure pump. The total flow rate is 3.3 µl s^−1^ (0.17 for the sample flow) and the total separation time is ~15–20 min for a sample of 200 µl. Experiments were done with 6 and 15 µm beads (Polybead® microspheres, Polysciences, Warrington, PA, USA), erythrocytes and boar spermatozoa. The erythrocytes and spermatozoa were imaged and counted for the fraction totals on a Nikon TE2000-U microscope (Nikon, Tokyo, Japan) equipped with a ×10 phase contrast objective. A high-speed camera (Photron SA-3, West Wycombe, UK) was mounted onto the microscope for image recording with the Photron software (Photron Fastcam Viewer). This high-speed camera imaged the cells passing through the transition from pinched to broadened section. Prior to each experiment, the chip was activated with oxygen plasma to prevent bubble formation during filling. The PDMS and glass surfaces of the chip were coated with poly(l-lysine)-grafted-poly(ethylene glycol) (PLL-g-PEG, SuSoS, Dübendorf, Switzerland) to prevent bead and cell adhesion during the separation experiments. PLL-g-PEG was rinsed through the PDMS micro channels at a concentration of 100 μg ml^−1^ in deionized (DI) water for at least 15 min. All experiments were carried out at room temperature to prevent swimming of the boar spermatozoa.

### Viability assay

The influence of the chip on the viability of the spermatozoa was assessed with a SYBR 14/propidium iodide (PI) live/dead staining. The spermatozoa were incubated in a 1000× dilution of SYBR 14 (stock 1 mM, ex/em 488/518 nm, Life Technologies) for 20 min and a 100× dilution of PI (stock 2.4 mM, ex/em 535/617 nm, Life Technologies) for 5 min at room temperature. To obtain the influence of the chip on the viability of the cells, the ratio of live/dead spermatozoa in the treated sample was divided by the ration of live/dead spermatozoa of the control sample.

### Particle distribution in the separation channel

A first set of experiments was done with 6 and 15 µm beads and boar spermatozoa, to characterize the particle distribution in the separation channel. A mixture of microspheres and spermatozoa was prepared and flown through the device to characterize the particle distribution in the channel. From this distribution, the preferred outlet flow rates were estimated and used in the erythrocytes/spermatozoa experiments. The particle distribution of erythrocytes was also characterized. This was done with a Matlab script, which counted and circled the beads/erythrocytes, after which the spermatozoa (and any beads/erythrocytes that were missed) were counted by hand. The positions relative to the channel wall were saved and plotted in a histogram.

### Separation of spermatozoa from erythrocytes

After determining the preferred flow rates for the spermatozoa and erythrocytes experiments, the separation of spermatozoa from the sample was characterized. For this, we defined three parameters.

1. Extraction purity (EP): the proportion of spermatozoa in the collection outlet, relative to the total number of cells extracted in this outlet. Therefore, EP reports the composition of the extracted sample.
2. Collection efficiency (CE): the proportion of spermatozoa or erythrocytes in the collection outlet, relative to the total number of that cell type in the sample. This gives an indication of the retention of the spermatozoa and the percentage of erythrocytes that cannot be separated in that same experiment.
3. Enrichment ratio (ER): the proportion of spermatozoa in the extraction outlet compared with the proportion at the inlet.

The flow ratio of buffer to sample in the inlet was 95:5 for each experiment, whereas the ratio of waste flow/total flow in the outlet was varied between 3% and 5%. To obtain the EP and CE, the spermatozoa and erythrocytes were counted in the inlet and each outlet with the same script as mentioned above, supplemented by manual counting. The ER was then calculated from the sample composition before and after separation.

### Simulations

Simulations have been performed with Comsol 5.1 (Comsol AB, Burlington, MA) to obtain simulation data for the position of 6 and 15 µm beads. The model uses fluid structure interaction in the pinched section, where the size of the bead (6 or 15 µm) is significant compared with the channel dimension (50 µm) to consider the effect that the particle has on the flow profile. Due to the large computation time of this method, the particle position after passing the pinched section was taken and further modeled with particle tracing to obtain the position over time in the broadened section. Particle tracing models the particle as just one point in the channel, with no major effects on the flow profile, which is now allowed due to the small size of the particle relative to the channel.

## Supplementary information

Video S1: High speed video of spermatozoa in pinch flow fractionation device

Video S2

Video S3

Supplemental Material

## References

[1] Gudeloglu A, Parekattil SJ (2013). Update in the evaluation of the azoospermic male. Clinics.

[2] Popal W, Nagy ZP (2013). Laboratory processing and intracytoplasmic sperm injection using epididymal and testicular spermatozoa: what can be done to improve outcomes?. Clinics.

[3] Parmegiani L (2010). “Physiologic ICSI”: hyaluronic acid (HA) favors selection of spermatozoa without DNA fragmentation and with normal nucleus, resulting in improvement of embryo quality. Fertil. Steril..

[4] Setti AS (2010). Intracytoplasmic sperm injection outcome versus intracytoplasmic morphologically selected sperm injection outcome: a meta-analysis. Reprod. Biomed. Online.

[5] Frimat J-P (2014). Make it spin: individual trapping of sperm for analysis and recovery using micro-contact printing. Lab Chip.

[6] De Wagenaar B (2015). Microfluidic single sperm entrapment and analysis. Lab Chip.

[7] de Wagenaar B (2016). Spermometer: electrical characterization of single boar sperm motility. Fertil. Steril..

[8] Hosokawa M (2012). Leukocyte counting from a small amount of whole blood using a size‐controlled microcavity array. Biotechnol. Bioeng..

[9] VanDelinder V, Groisman A (2007). Perfusion in microfluidic cross-flow: separation of white blood cells from whole blood and exchange of medium in a continuous flow. Anal. Chem..

[10] Son J (2015). Non-motile sperm cell separation using a spiral channel. Anal. Methods.

[11] Liu W (2015). Separation of sperm and epithelial cells based on the hydrodynamic effect for forensic analysis. Biomicrofluidics.

[12] Diez-Silva M (2010). Shape and biomechanical characteristics of human red blood cells in health and disease. MRS Bull..

[13] Bonet, S. et al. *Boar Reproduction: Fundamentals and New Biotechnological Trends* (Springer, Berlin Heidelberg, 2013).

[14] World Health Organization *WHO Laboratory Manual for the Examination and Processing of Human Semen* (World Health Organization, Geneva, 2010).

[15] Samuel R (2016). Microfluidics: the future of microdissection TESE?. Syst. Biol. Reprod. Med..

[16] Mishra, Y.N. et al. A microfluidic device for the study of the orientational dynamics of microrods. In *Proceedings of SPIE - The International Society for Optical Engineering*. **8251**, 825109 (2012). 10.1117/12.915871.

[17] Kaya T, Koser H (2009). Characterization of hydrodynamic surface interactions of *Escherichia coli* cell bodies in shear flow. Phys. Rev. Lett..

[18] Dupire J, Socol M, Viallat A (2012). Full dynamics of a red blood cell in shear flow. Proc. Natl Acad. Sci. USA.

[19] Sugaya S, Yamada M, Seki M (2011). Observation of nonspherical particle behaviors for continuous shape-based separation using hydrodynamic filtration. Biomicrofluidics.

[20] Uspal WE, Burak Eral H, Doyle PS (2013). Engineering particle trajectories in microfluidic flows using particle shape. Nat. Commun..

[21] Bretherton F, Rothschild NMV (1961). Rheotaxis of spermatozoa. Proc. R. Soc. Lond. B.

