# Supplementary material for: Separation of spermatozoa from erythrocytes using their tumbling mechanism in a pinch flow fractionation device
Source: Microsyst Nanoeng. 2019 May 20;5:24. doi: 10.1038/s41378-019-0068-z (PMC6527678; doi:10.1038/s41378-019-0068-z)
Supplement: Supplementary file 4 — Supplemental Material [file 41378_2019_68_MOESM4_ESM.docx]

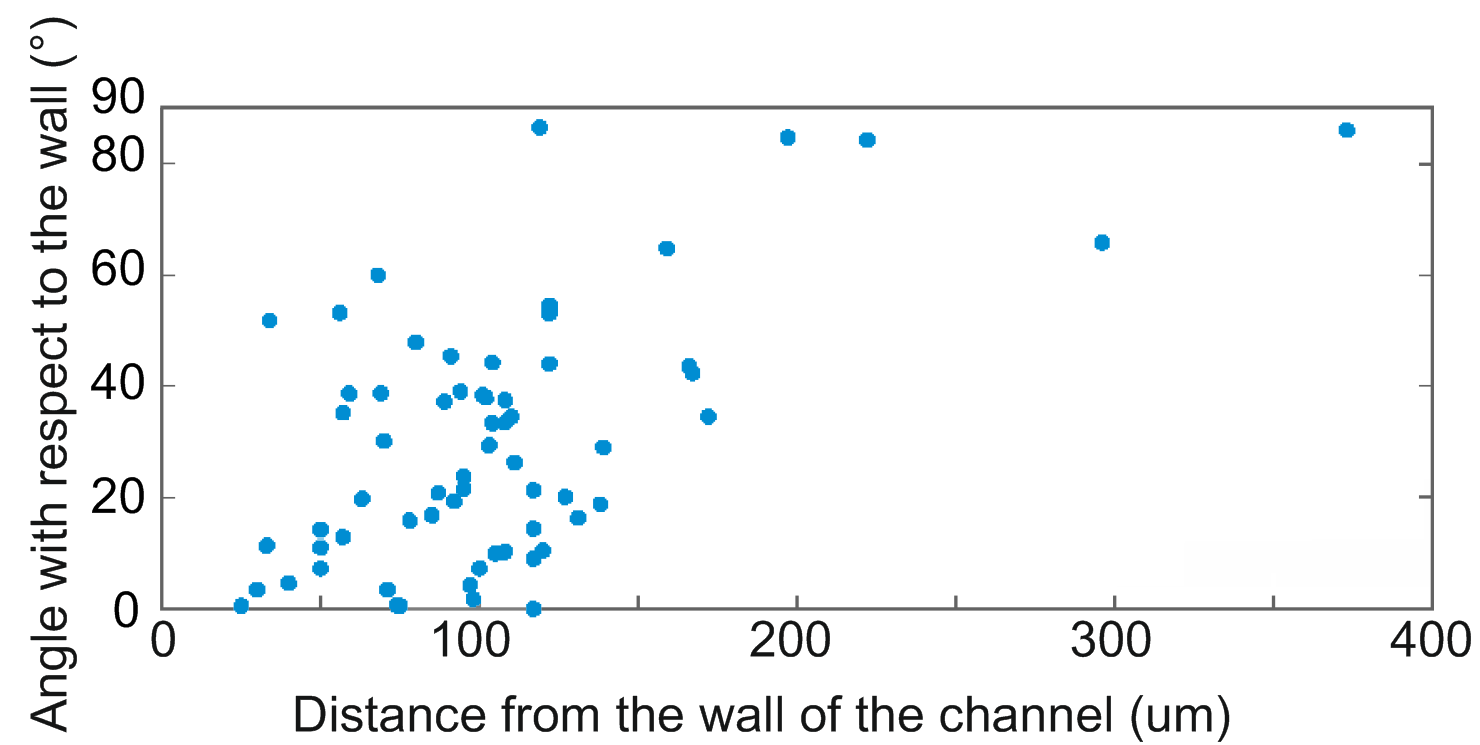


Figure S1: Angle of the spermatozoa with respect to the wall as a function of their distance. A correlation can be seen. A larger angle seems to result on average in a larger distance from the wall in the broadened channel

Video S1: Several spermatozoa passing through the transition from the pinched section to the broadened section. The shear in the fluid causes them to rotate. Video was taken at 10.000 fps and played back 345 times slower.

Video S2: Many erythrocytes and a spermatozoon pass through the transition from the pinched section to the broadened section. The spermatozoon is indicated with an arrow and passes at a larger distance from the side wall than the erythrocytes. Video was taken at 1.000 fps and played back 67 times slower.

Video S3: Several spermatozoa and erythrocytes in the broadened section. The spermatozoa have a larger distance from the side wall, although the positions of the two types of cells overlap slightly. Video was taken at 1.000 fps and played back 34.5 times slower.


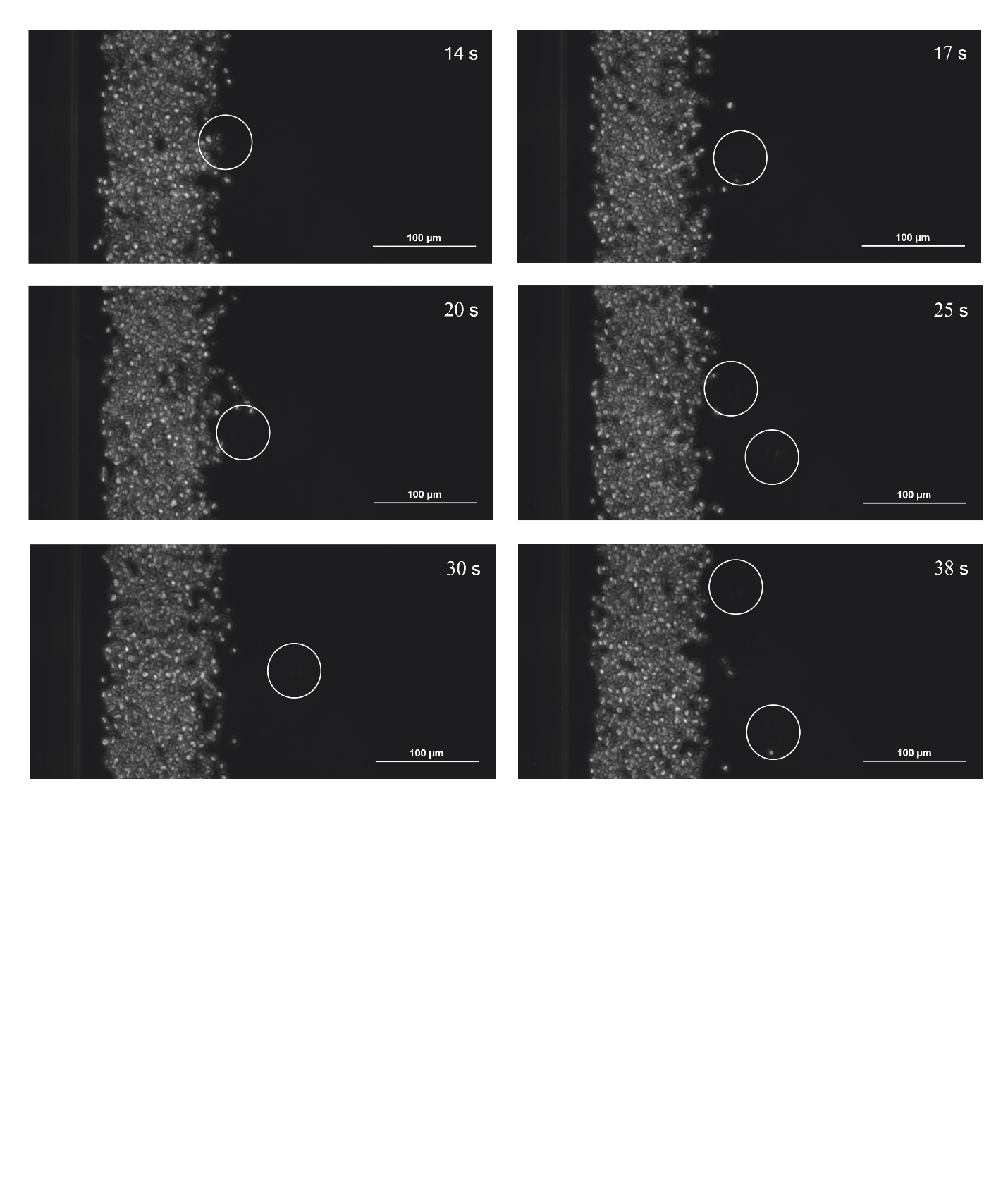


Figure S2: Stills from Video S3 (timestamp in upper right corner). The spermatozoa are indicated with a circle.
